# Supplementary material for: Photochemical Consideration in the Interactions between Blood Proteins and Layered Inorganic Materials
Source: Int J Mol Sci. 2022 Sep 26;23(19):11367. doi: 10.3390/ijms231911367 (PMC9570392; doi:10.3390/ijms231911367)
Supplement: Supplementary file 1 [file ijms-23-11367-s001.zip › ijms-1915930-supplementary.pdf]

Supporting Information for:

Photochemical consideration on the interactions between blood proteins and layered inorganic materials

Tetsuo Yamaguchi[1], Hyoung-Mi Kim[2], Jae-Min Oh[1*]

[1]*Department of Energy and Materials Engineering, Dongguk University-Seoul, 04620, Seoul, Korea*

[2]Biomedical Manufacturing Technology Center, Korea Institute of Industrial Technology (KITECH), Daegyeong Division, 59, Yangho-gil, Yeongcheon-si, Gyeongsangbuk-do, 38822 Republic of Korea

[*] Correspondence should be addressed to J. M. Oh.
E-mail: jaemin.oh@dongguk.edu
Tel: +82-2-2260-4977

1. Powder XRD patterns
2. Zeta potential
3. Absorption spectra
4. Fluorescence spectra
5. Stern-Volmer plots

1. Powder XRD patterns

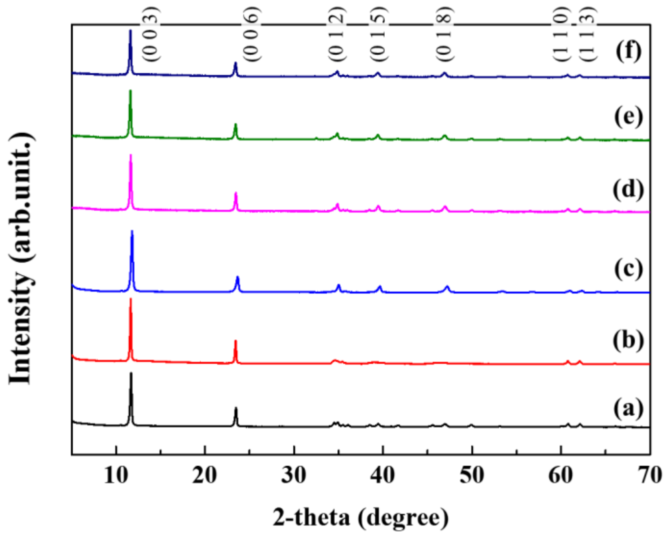

Figure S1. Powder X-ray diffraction patterns for (a) LDH-S, (b) LDH-M, (c) LDH-L, (d) LDH-(−), (e) LDH-(0) and (f) LDH-(+).

2. Zeta potential

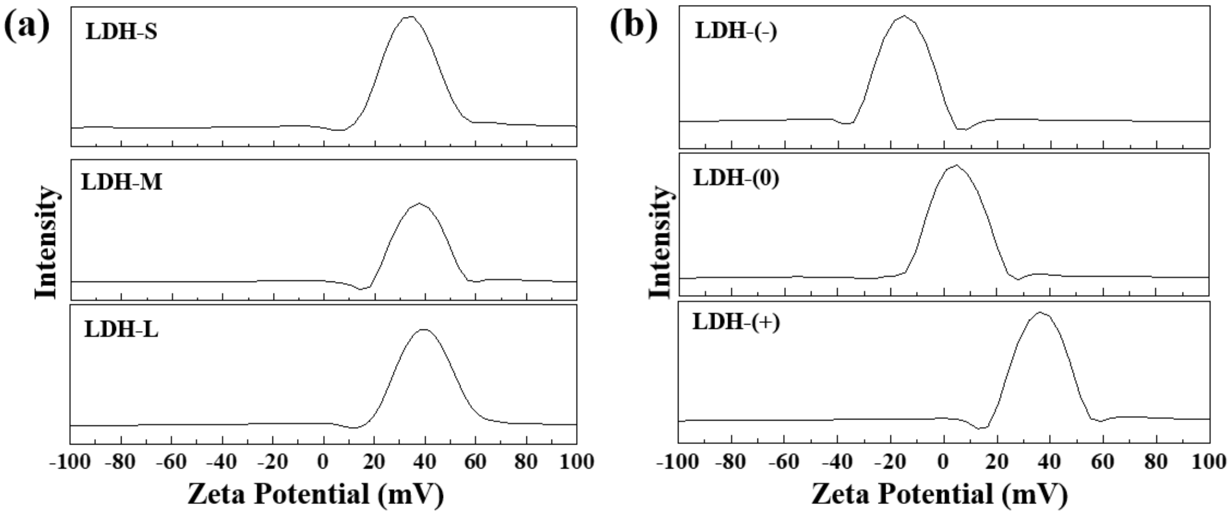

Figure S2. Zeta potential values of LDHs (a) in different sizes and (b) in different surface charges.

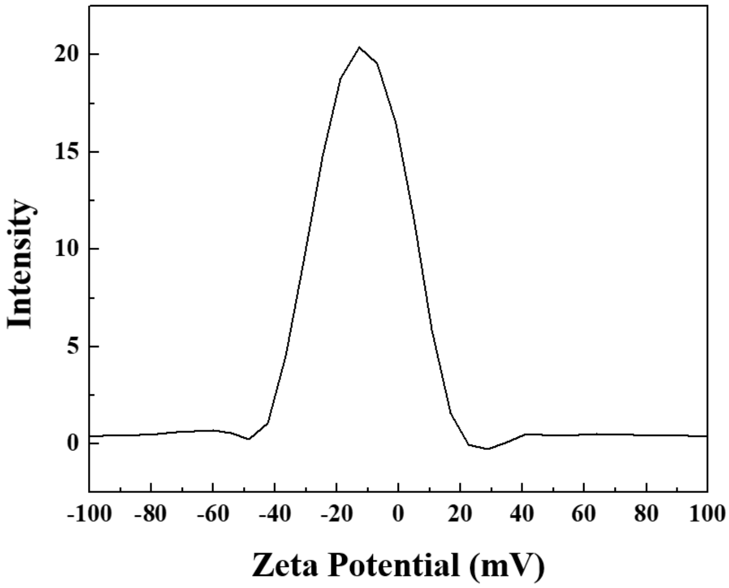

Figure S3. Zeta potential value of human whole blood 10 times diluted with phosphate buffered saline (PBS).

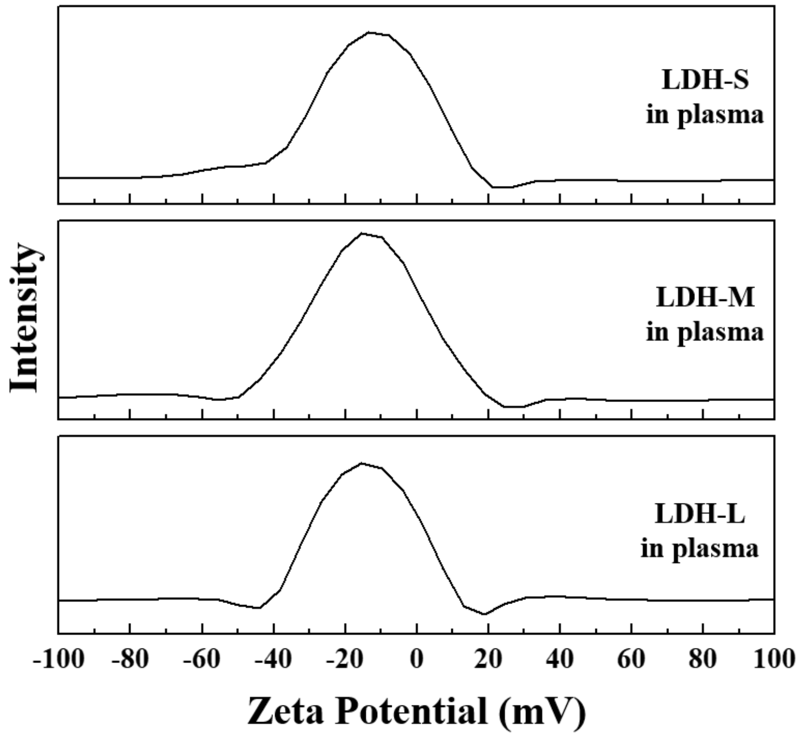

Figure S4. Zeta potential values for the LDH nanoparticles treated with human blood plasma.

3. Absorption spectra

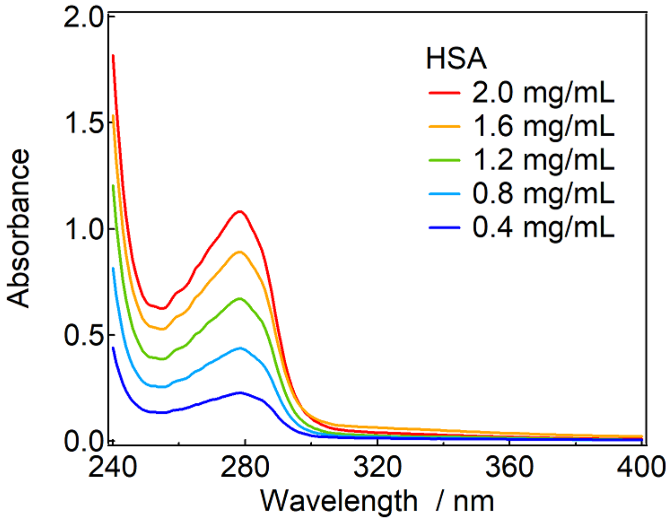

Figure S5. Absorption spectra of HSA at several concentrations for adsorption isotherms.

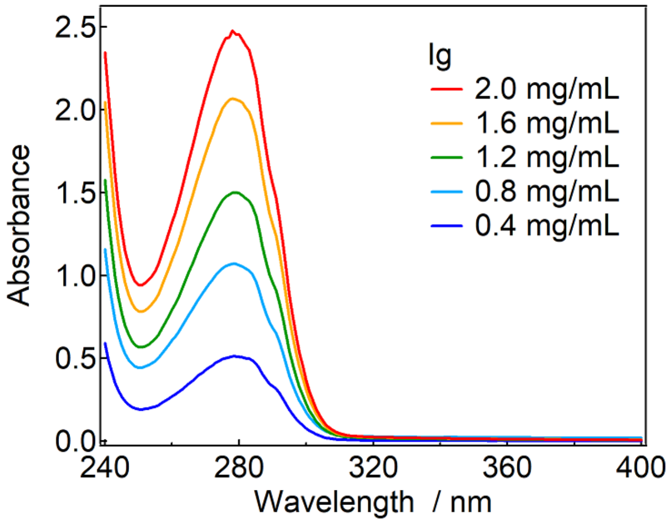

Figure S6. Absorption spectra of Ig at several concentrations for adsorption isotherms.

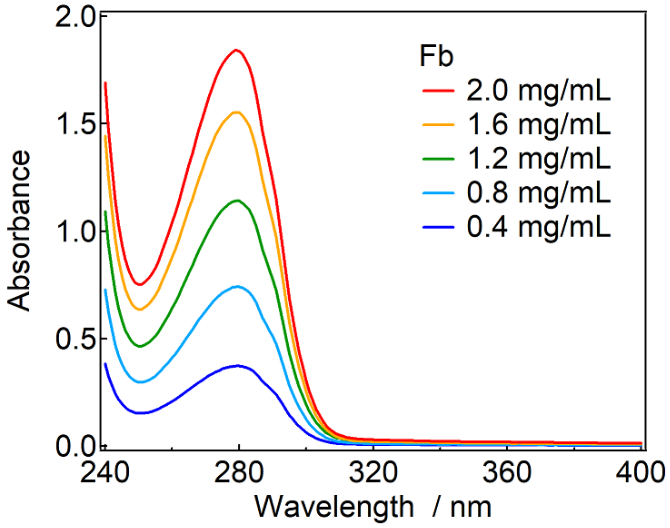

Figure S7. Absorption spectra of Fb at several concentrations for adsorption isotherms.

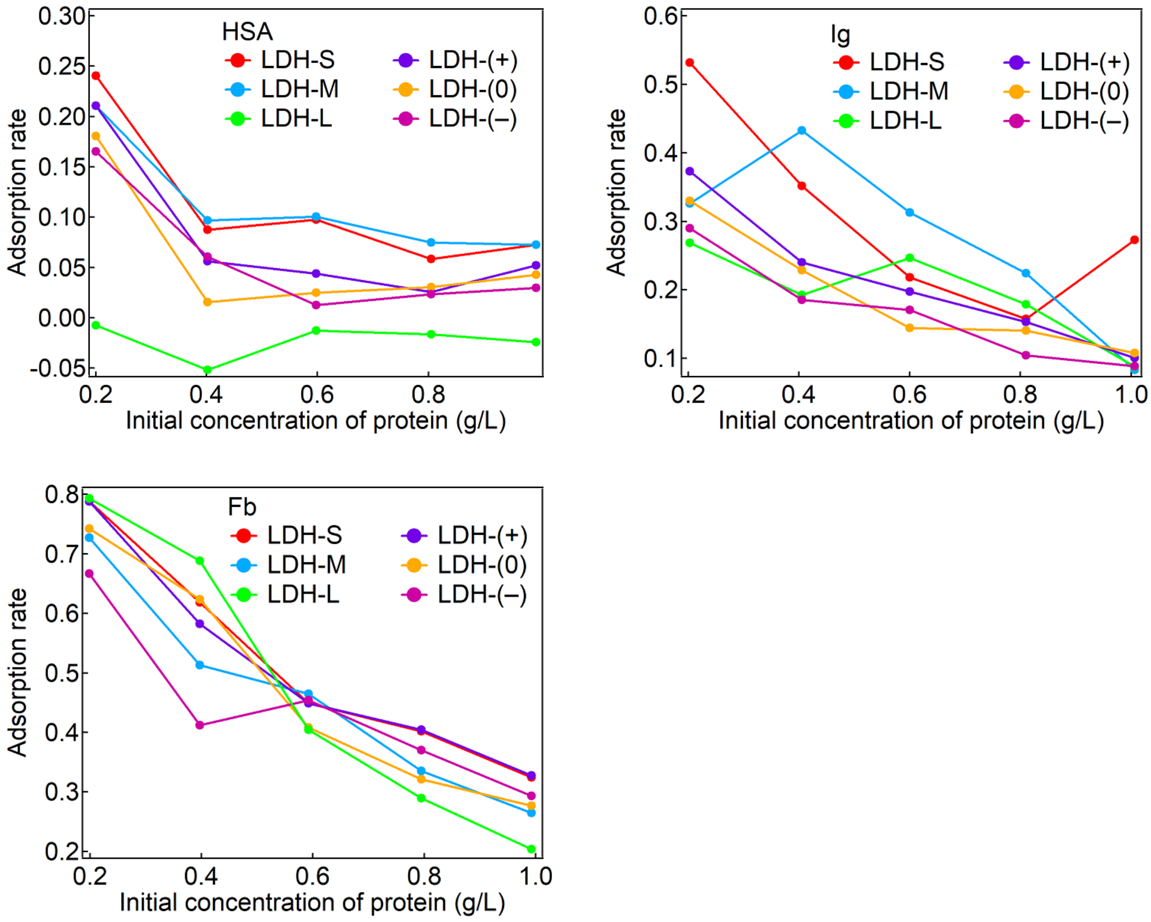

Figure S8. Adsorption rate of proteins on to the LDHs at concentration of 5 g-LDH/L.

4. Fluorescence spectra

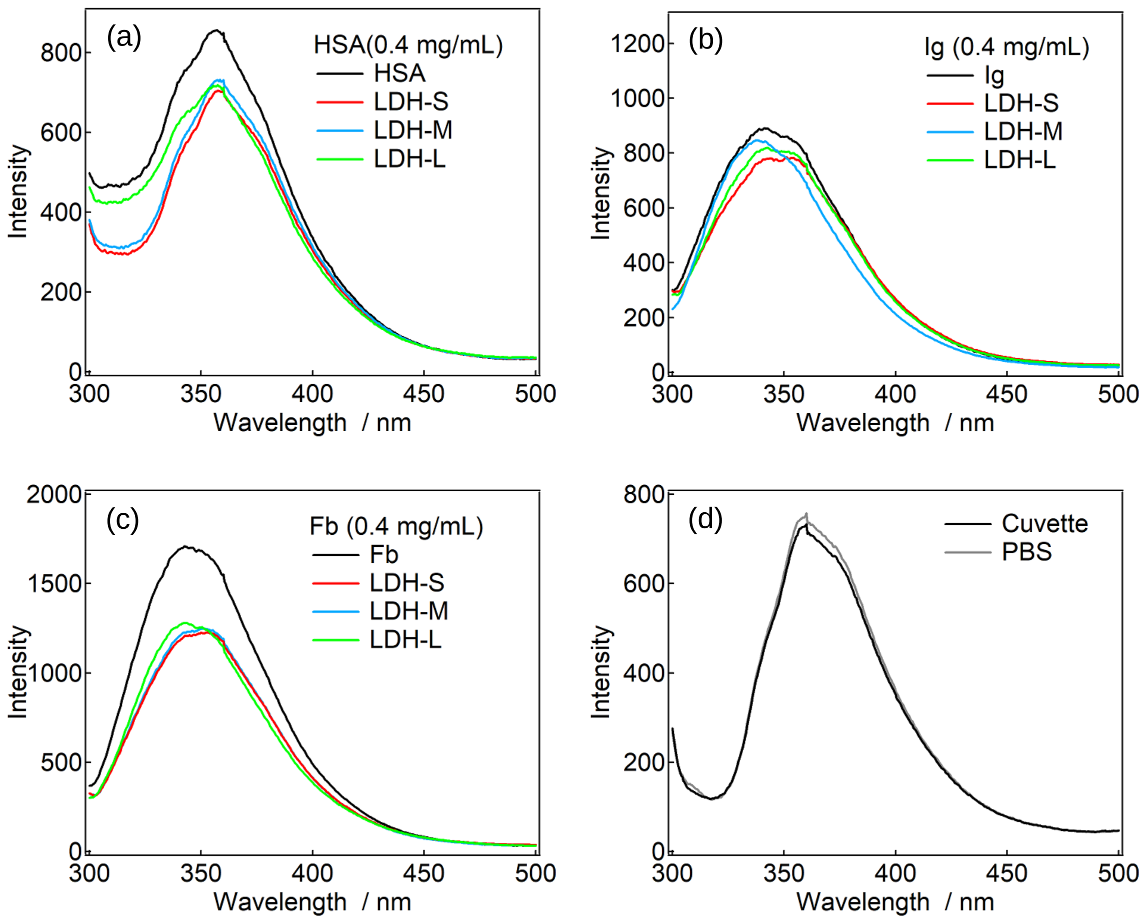

Figure S9. Emission spectra of (a) HSA, (b) Ig and (c) Fb in PBS (black line), in LDH-S (red line), LDH-M (blue line) and LDH-L suspensions (green line), and emission spectra of a quartz cuvette with/without PBS.

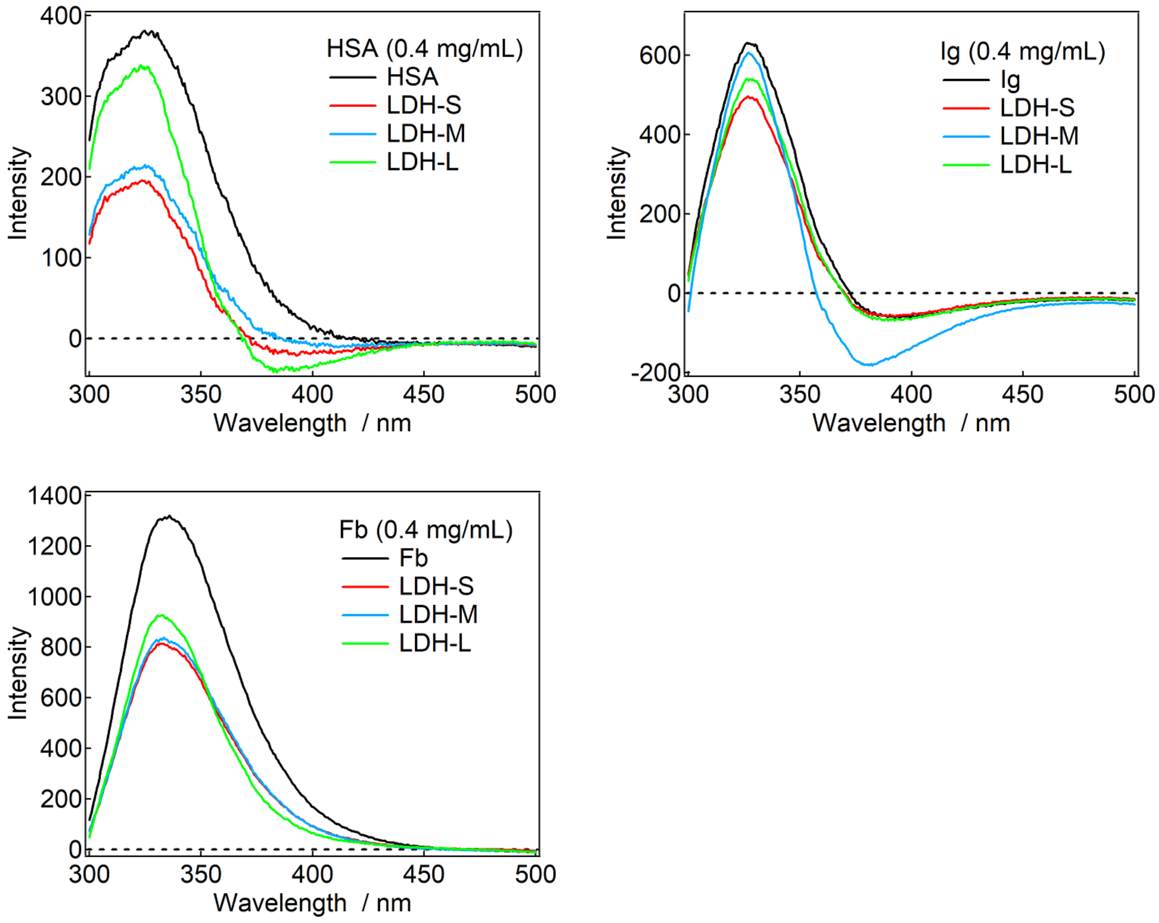

Figure S10. Emission spectra of (a) HSA, (b) Ig and (c) Fb in PBS (black line), in LDH-S (red line), LDH-M (blue line) and LDH-L suspensions (green line) after subtraction of the emission of the quartz cuvette.

5. Stern-Volmer plots

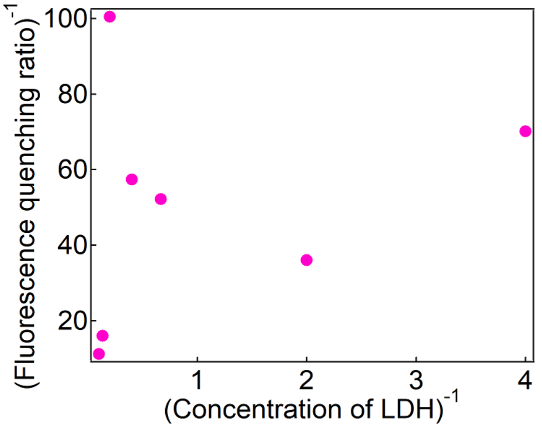

Figure S11. Modified Stern-Volmer plot for fluorescence quenching for blood plasma by LDH-(−).

Table S1 Fitting parameter for fluorescence quenching of blood plasma proteins obtained from fitting with the equation (4) which is Stern-Volmer plot with three fluorophores in main manuscript.

	$K_{SV}(1)$	$K_{SV}(2)$	$f(1)$	$f(2)$	$f(3)$
LDH-S	5.63	−0.000145	0.260	0.120	0.621
LDH-M	5.97	0.0153	0.244	0.126	0.631
LDH-L	1.83	−0.00834	0.313	0.294	0.403
LDH-S(−)	0.0200	0.0218	0.200	0.200	0.595
LDH-S(0)	18.4	0.00885	0.214	0.436	0.350
LDH-S(+)	3.29	−0.00614	0.316	0.138	0.545

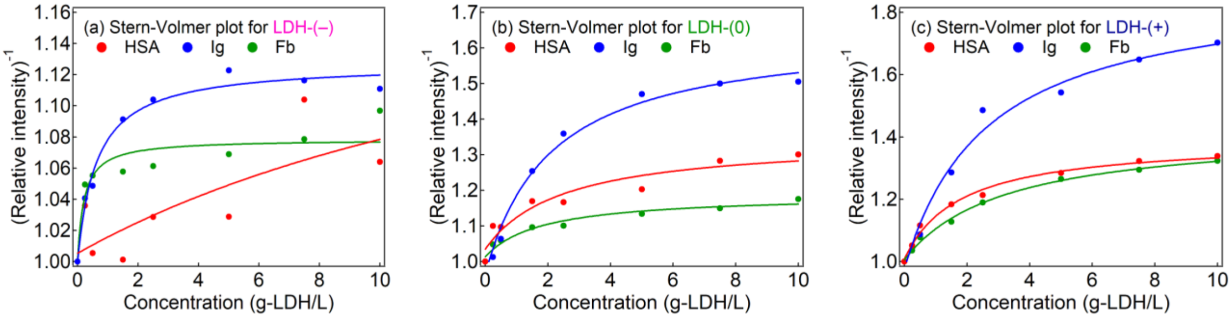

Figure S12. Stern-Volmer plots for fluorescence quenching of the three proteins by (a) LDH-S(−), (b) LDH-(0) and (c) LDH-(+). Solid lines: fitting lines by equation (5) in main manuscript.

Table S2. Fitting parameters obtained from Stern-Volmer plots shown in Fig. S12 by fitting with equation (5) in main manuscript.

LDHs	protein	$K_{SV}(1)$	$f(1)$	$f(2)$
LDH-S(−)	HSA	0.0605	0.179	0.816
	Ig	1.80	0.113	0.887
	Fb	4.95	0.0715	0.927
LDH-S(0)	HSA	0.526	0.222	0.774
	Ig	0.815	0.430	0.606
	Fb	0.647	0.145	0.842
LDH-S(+)	HSA	0.771	0.272	0.719
	Ig	0.729	0.494	0.529
	Fb	0.441	0.291	0.703

Table S3. Fitting parameters obtained from modified Stern-Volmer plots of three types of proteins by surface charge controlled LDHs.

LDHs	protein	$K_{SV}(1)$ (L(g-LDH) $^{-1}$)	τ_0 (ns)	k_q (L(g-LDH)$^{-1}$ s^{-1})	$f(1)$
LDH-S(−)	HSA	N.A.	7.8[a]	N.A.	N.A.
	Ig	2.059	4.65[b]	4.43×10^{8}	0.107
	Fb	N.A.	2.1[c]	N.A.	N.A.
LDH-S(0)	HSA	N.A.	7.8[a]	N.A.	N.A.
	Ig	N.A.	4.65[b]	N.A.	N.A.
	Fb	N.A.	2.1[c]	N.A.	N.A.
LDH-S(+)	HSA	0.888	7.8[a]	1.14×10^{8}	0.283
	Ig	0.374	4.65[b]	8.04×10^{7}	0.563
	Fb	0.570	2.1[c]	2.71×10^{8}	0.292

[a]used longest life-time component [1], [b]used longest life-time component [2], [c]as F-actin[3]

References

(1) Beechem, J. M.; Brand, L. Time-Resolved Fluorescence of Proteins. *Annu. Rev. Biochem.* **1985**, *54*, 43–71.

(2) Demeule, B.; Lawrence, M. J.; Drake, A. F.; Gurny, R.; Arvinte, T. Characterization of Protein Aggregation: The Case of a Therapeutic Immunoglobulin. *Biochim. Biophys. Acta - Proteins Proteomics* **2007**, *1774* (1), 146–153. https://doi.org/10.1016/j.bbapap.2006.10.010.

(3) Burstein, E.; Vedenkina, N.; Ivkova, M. Fluorescence and the Location of Tryptophan

Residues in Protein Molecules. *Photochem. Photobiol.* **1973**, *18*, 263–279.
